# Dexmedetomidine Alleviates Lipopolysaccharide-Induced Acute Kidney Injury by Inhibiting p75NTR-Mediated Oxidative Stress and Apoptosis

**DOI:** 10.1155/2020/5454210

**Published:** 2020-10-31

**Authors:** Zhe Wang, Jiali Wu, Zhaolan Hu, Cong Luo, Pengfei Wang, Yanling Zhang, Hui Li

**Affiliations:** ^1^Department of Anesthesiology, The Second Xiangya Hospital, Central South University, Changsha, China; ^2^Department of Laboratory Medicine, The First Affiliated Hospital, Sun Yat-sen University, Guangzhou, China

## Abstract

Oxidative stress and apoptosis play a key role in the pathogenesis of sepsis-associated acute kidney injury (AKI). Dexmedetomidine (DEX) may present renal protective effects in sepsis. Therefore, we studied antioxidant effects and the mechanism of DEX in an inflammatory proximal tubular epithelial cell model and lipopolysaccharide- (LPS-) induced AKI in mice. *Methods*. We assessed renal function (creatinine, urea nitrogen), histopathology, oxidative stress (malondialdehyde (MDA) and superoxide dismutase (SOD)), and apoptosis (TUNEL staining and Cleaved caspase-3) in mice. *In vitro* experiments including Cleaved caspase-3 and p75NTR/p38MAPK/JNK signaling pathways were evaluated using western blot. Reactive oxidative species (ROS) production and apoptosis were determined using flow cytometry. *Results*. DEX significantly improved renal function and kidney injury and also revert the substantially increased level of MDA concentrations as well as the reduction of the SOD enzyme activity found in LPS-induced AKI mice. In parallel, DEX treatment also reduced the apoptosis and Cleaved caspase-3 expression evoked by LPS. The expression of p75NTR was increased in kidney tissues of mice with AKI but decreased after treatment with DEX. In cultured human renal tubular epithelial cell line (HK-2 cells), DEX inhibited LPS-induced apoptosis and generation of ROS, but this was reversed by overexpression of p75NTR. Furthermore, pretreatment with DEX significantly downregulated phosphorylation of JNK and p38MAPK in LPS-stimulated HK-2 cells, and this effect was abolished by overexpression of p75NTR. *Conclusion*. DEX ameliorated AKI in mice with sepsis by partially reducing oxidative stress and apoptosis through regulation of p75NTR/p38MAPK/JNK signaling pathways.

## 1. Introduction

Sepsis is a complex disease characterized by a maladaptive host response to infection resulting in organ dysfunction and shock [1]. More than 50% of patients with sepsis develop acute kidney injury (AKI), a common complication of sepsis and endotoxemia [2]. Lipopolysaccharide (LPS) is a component of the outer membrane of Gram-negative bacteria, and an injection of LPS is commonly used in a model of experimental sepsis-associated AKI [3]. LPS primarily combines with toll-like receptor 4 (TLR4) to activate an inflammation pathway [4] and simultaneously generate abundant reactive oxygen species (ROS) [5]. ROS triggers cellular dysfunction, detachment, and apoptotic cell death in proximal tubules [6]. Studies have reported that sepsis-related AKI is principally caused by glomerular or tubular apoptosis, in particular in tubular epithelial cells [7]. Little progress has been made on the pharmacological treatment of AKI associated with sepsis, despite the existing knowledge on the prevention mechanism of oxidative stress and apoptosis in proximal tubular cells which is critical to the management of sepsis-associated AKI.

The *α*_2_-adrenoreceptors are widely distributed in renal proximal and distal tubules and in peritubular tissues [8]. Dexmedetomidine (DEX) is a potent *α*_2_-adrenergic agonist, which has been proclaimed to exhibit antioxidant and anti-inflammatory effects [9, 10]. Previous research has demonstrated that DEX mitigates apoptosis; it has been established to protect organs by exhibiting protective effects against ischemia/reperfusion (I/R) injury in the liver, heart, and kidney [11–13]. Intraperitoneal injection of DEX was able to alleviate oxidative stress damage in LPS-induced acute liver injury [14]. Of particular importance is the protective effect of DEX on perioperative AKI revealed in several clinical studies [9, 15]. Experimental studies have demonstrated that DEX remarkably attenuates renal oxidative stress and apoptosis in early LPS-induced AKI [16]. However, the exact effect of DEX on tubular epithelial cells in mice with sepsis-associated AKI has not been fully elucidated.

The p75 neurotrophin receptor (p75NTR) is a multifunctional transmembrane protein with the ability to bind members of the neurotrophin family, including nerve growth factor (NGF), brain-derived neurotrophic factor (BDNF), and their precursors like proNGF and proBDNF [17]. Studies of mice have revealed that p75NTR is crucial for naturally occurring developmental apoptosis within the retina, superior cervical ganglia, spinal cord, and basal forebrain [18–21]. A lipid peroxidation product, 4-hydroxynonenal (HNE), resulted in neurite degeneration and apoptosis, which was reduced in p75NTR^−/−^ mice [22]. Moreover, several types of injury such as seizure, ischemia, and oxidative stress cause upregulation of p75NTR in brain neurons [23, 24]. p75NTR-induced superoxide production and subsequent apoptosis have also been reported in motor neurons [25]. p75NTR has also been reported to participate in kidney development and is distributed in kidney tissues [26]. Strikingly, p75NTR is overexpressed in kidneys under pathological conditions such as renal cell carcinoma [27], diabetic nephropathy [28], and chronic kidney disease [29].

No study has focused on the expression and role of p75NTR in AKI to date. Protective effects of DEX in high glucose-induced apoptosis can be reversed by overexpression of p75NTR in human retinal pigment epithelial cells [30], suggesting that DEX acts on p75NTR in AKI. Therefore, we examined whether DEX can alleviate LPS-induced apoptosis in tubular epithelial cells and AKI and the role of p75NTR.

## 2. Materials and Methods

### 2.1. Animals and Treatments

Male C57BL/6 mice (age, 7-8 weeks; weight, 20–22 g) were obtained from the Central South University Animal Service (Changsha, China). Mice were housed under conditions of constant temperature (25°C), 50 ± 10% relative humidity, and 12-hour light-dark cycle, and they had free access to food and water. Mice were randomly divided into the following three groups (*n* = 6–10 in each group): (I) control group mice i.p. with saline solution, (II) LPS group mice i.p. with LPS (L2880-25MG, Sigma-Aldrich, St. Louis, CA, USA) at a dose of 10 mg/kg once, and (III) LPS+low dose of DEX group which had mice i.p. injected with 10 *μ*g/kg of DEX (H20090248, Jiangsu Hengrui Pharmaceutical Co., Ltd., China) 30 minutes before treatment with LPS, (IV) LPS+DEX group which had mice i.p. with 30 *μ*g/kg of DEX 30 minutes before treatment with LPS, and (V) LPS+large dose of DEX group which had mice i.p. with 50 *μ*g/kg of DEX 30 minutes before treatment with LPS. The mice in each group had free access to food and water under pathogen-free conditions. After LPS administration, survival rate was then recorded for the next 120 hours. All experiments were approved by the Hospital Ethics Committee of the Second Xiangya Hospital of Central South University and carried out in accordance with the National Institutes of Health Guide for the Care and Use of Laboratory Animals (NIH Publications No. 8023, revised 1978).

### 2.2. Cell Culture and Treatments

Human renal tubular epithelial cell line (HK-2 cell) was purchased from the Cell Bank of the Chinese Academy of Sciences and cultured in minimum essential medium (MEM) (Invitrogen, Carlsbad, CA, USA) with 10% fetal bovine serum (FBS) (Invitrogen, Carlsbad, CA, USA) at a temperature of 37°C and 5% CO_2_. In the *in vitro* experiments and western blot of the signaling pathway, the dose of LPS and DEX is as follows: LPS: HK-2 cells (density, 1 × 10^5^ cells per well) in 12-well plates were treated with 1 *μ*g/mL of LPS; LPS+DEX: HK-2 cells were treated with 1 *μ*g/mL of LPS and 100 *μ*M of DEX for 24 hours. All groups are treated for 24 hours.

### 2.3. p75NTR Overexpression Assay

pc/p75, a pcDNA3.1 expression vector inserted in the human p75NTR gene, was purchased from GeneChem (Shanghai, China). pc/C, an empty pcDNA3.1, was also purchased from GeneChem (Shanghai, China) and used as control overexpression vector. HK-2 cells were transfected with pc/p75 or pc/C using Lipofectamine 3000 (Invitrogen, Shanghai, China) according to the manufacturer's method; the effect of p75NTR overexpression was analyzed by western blot assay after transfection.

### 2.4. Kidney Function Test

Mice were sacrificed at 24 hours after LPS injection, and serum was collected. Whole blood was drawn and immediately anticoagulated with ethylenediaminetetraacetic acid- (EDTA-) 2K. Subsequently, serum creatinine (Cr) and blood urea nitrogen (BUN) were automatically detected using automatic cell analyzers (ARCHITECT c 8000, Abbott Corporation, Chicago, USA).

### 2.5. Reverse Transcription and Quantitative Real-Time Polymerase Chain Reaction (PCR)

Mice were sacrificed at 24 hours after LPS injection; kidney tissues were collected. Total RNA was extracted from kidney tissues as described previously [31]. Complementary DNA (cDNA) was synthesized using RevertAid First-Strand cDNA Synthesis kits (Thermo Scientific, Waltham, USA). Quantitative real-time PCR was performed using Synergy Brands (SYBR) Green (Bio-Rad, Hercules, CA, USA) on CFX96 Touch™ Deep Well Real-Time PCR Detection System (Bio-Rad, Hercules, CA, USA). PCR primers were ACCACCATGGAGAAGGCTGG and CTCAGTGTAGCCCAGGATGC (glyceraldehyde-3-phosphate dehydrogenase (GAPDH)); GACAGCACAGAATGTTCCAG and TGGCCAGATGTTCCTCTATT (inducible NOS (iNOS)); AGGGCACATACTCAGACGAA and AGATGGAGCAATAGACAGGAAT (p75NTR); ACCAACAATACGCACCAGC and AATAGCCATGCCGAACTCC (Sortilin); TCATAAGATCCCCCTGGATG and TGCTTCTCAGCTGCCTGAC (tyrosine kinase receptor A (TrkA)); CAACAGGACTCACCGGAGCA and GGCTGCAGGCAAGTCAGCCT (NGF). Quantitative real-time PCR was performed as per the manufacturer's instructions: 95°C for 3 min, 40 cycles of 95°C for 10 sec, and 59°C for 30 s. The experiment was conducted in triplicate. Data were processed using the 2^(-*ΔΔ*Ct)^ method.

### 2.6. Oxidative Stress Evaluation

Mice were sacrificed at 24 hours after LPS injection; kidney tissues were collected. The kidney tissues were homogenized with phosphate-buffered solution (PBS), and the supernatants were collected. Then, the levels of malondialdehyde (MDA) and superoxide dismutase (SOD) enzyme activity were determined using commercial detection kits (A003-1 and A001-1-1, Nanjing KeyGen Biotech. Co. Ltd., Nanjing, China) according to the manufacturer's instructions. ROS of cells was assessed by flow cytometry using total reactive oxygen species (ROS) assay kit 520 nm (88-5930-74; Invitrogen, Carlsbad, CA, USA). Cells were read on the flow cytometer (Cytek, Fremont, CA, USA), and data were analyzed with the FlowJo vX0.7 software. All the steps were performed according to the manufacturer's instructions.

### 2.7. Measurement of Apoptosis

Mice were sacrificed at 24 hours after LPS injection; kidney tissues were collected. Kidneys from mice were fixed and made into paraffin sections. Apoptosis in kidney tissues was analyzed using a TUNEL assay kit (Roche Diagnostics, Indianapolis, USA) according to the instructions of the manufacturer. Five high-power fields (×200) were randomly selected from each slice, the number of apoptotic cells and the total number of cells were counted, and the apoptosis index (AI) = the number of apoptotic cells/the total number of cells × 100%. The HK-2 cells were assessed by flow cytometry using an Annexin V-FITC/PI kit (556547, Becton Dickinson, USA). All the steps were performed according to the manufacturer's instructions.

### 2.8. Western Blot

Mice were sacrificed at 24 hours after LPS injection; kidney tissues were collected. Kidney tissues were lysed in a radioimmunoprecipitation assay lysis buffer (CW Biotech, Jiangsu, China) with 1% protease inhibitor cocktails (Sigma-Aldrich, St. Louis, CA, USA) and 1% EDTA solution. Concentration of proteins was evaluated using bicinchoninic acid (BCA) protein assay kit (CW Biotech, Wuhan, China). Proteins were separated using electrophoresis and then transferred onto polyvinylidene fluoride (PVDF) membranes. Proteins were blocked with PBS and 10% fat-free milk for 1 hour followed by incubation overnight at 4°C with the following primary antibodies: anti-proNGF, PA5-77532 (1 : 5000, Sigma-Aldrich, St. Louis, CA, USA); anti-p75 antibody, ab8874 (1 : 5000); anti-NGF antibody, ab52918 (1 : 500, Abcam, Cambridge, United Kingdom); anti-p-JNK, ab124956 (1 : 2500, Abcam, Cambridge, United Kingdom); anti-JNK, ab179461 (1 : 2500, Abcam, Cambridge, United Kingdom); anti-GAPDH antibody, ab8245 (1 : 5000, Abcam, Cambridge, United Kingdom); anti-Cleaved caspase-3 antibody, #9661 (1 : 1000, Cell Signaling Technology, Boston, USA); anti-P-p38, #4511 (1 : 2000, Cell Signaling Technology, Boston, USA); and anti-p38, #8690 (1 : 2000, Cell Signaling Technology, Boston, USA). Blot was then incubated with horseradish peroxidase-conjugated goat anti-rabbit IgG or goat anti-mouse IgG in Tris-buffered saline (TBS) for 2 hours at room temperature. The PVDF membrane was then exposed to film before development. Western blot band was analyzed using the mean grey value with NIH ImageJ program version 7.0 and standardized to GAPDH.

### 2.9. Histological Analysis

Mice were sacrificed at 24 hours after LPS injection; kidney tissues were collected. We fixed kidney tissues with 4% paraformaldehyde for 48 hours and subsequently subjected them to paraffin embedding. Mouse kidney was fixed, prepared into paraffin sections, and then stained with either hematoxylin and eosin (H&E) or Periodic Acid-Schiff stain (PAS). All images were viewed under a microscope (Nikon ECLIPSE 80i, Nikon Corporation, Tokyo, Japan) and analyzed using the ImageJ software version 7.0 (Media Cybernetics, Rockville, USA). Two pathologists used the Jablonski semiquantitative score to evaluate the degree of renal tubular damage 0-4 points: 0 points: normal; 1 point: the most mild injury of cortical or medullary outer layer bad < 5%; 2 points: 5%-25% of leather or medullary outer layer injury; 3 points: 26%-75% of leather or medullary outer layer injury; 4 points: >75% of leather or medullary outer layer injury [32].

### 2.10. Immunohistochemistry (IHC) Analysis

Paraffin-embedded tissues were cut into 4 *μ*m thick sections, followed by antigen unmasking process, and incubated overnight at 4°C with Cleaved caspase-3 antibody (1 : 1000, #9661, Cell Signaling Technology, Boston, USA). Phosphate-buffered saline replaced the primary antibody as a negative control. The subsequent detection was done with the use of anti-rabbit or mouse immunohistochemistry assay kit (DAKO, Carpentaria, CA, USA) as a chromogen for visualization. Finally, hematoxylin was used to counterstain the nuclei. We chose five 40x magnification fields per tissue section at random, and two independent blinded observers obtained the mean area values of positive signals for final analysis by using the ImageJ software version 7.0 (Media Cybernetics, Rockville, USA).

### 2.11. Statistical Analysis

Data are expressed as mean ± standard error of the mean (SEM). Statistical analysis was performed using the paired Student's *t*-test or one-way analysis of variance (ANOVA) followed by Bonferroni analysis where appropriate. Statistical significance was arbitrarily declared at *p* values below 0.05. All analyses were performed using SPSS version 23 (SPSS Inc., Chicago, IL, USA).

## 3. Results

### 3.1. DEX Alleviates LPS-Induced AKI

**Figure 1:**
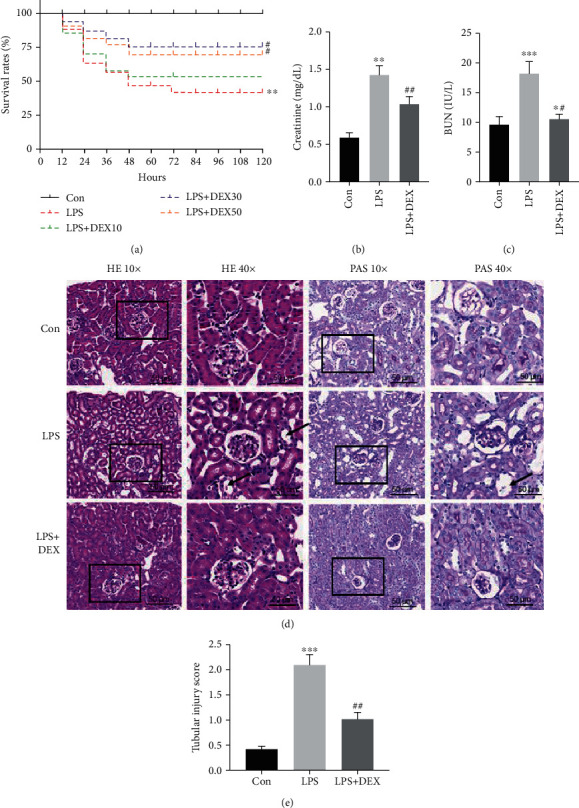
DEX improves survival rate and attenuates kidney damage induced by LPS. (a) Survival rates of septic mice, 10, 30, and 50 *μ*g/kg DEX pretreatment groups (^∗∗^*p* < 0.01, versus control; ^#^*p* < 0.05 versus LPS, *n* = 10 per group; log-rank (Mantel-Cox) test). (b, c) Cr and BUN levels in serum. (d) Histopathological changes in kidney tissues (scale bar = 50 *μ*m); yellow arrow indicates tubular necrosis and vacuolar degeneration. (e) Tubular injury score (^∗^*p* < 0.05, ^∗∗^*p* < 0.01, and ^∗∗∗^*p* < 0.0001 versus control; ^##^*p* < 0.01 versus LPS. Data were presented as mean ± SEM, *n* = 6). H&E: hematoxylin and eosin; PAS: Periodic Acid-Schiff stain.

As shown in Figure 1(a), survival rate dropped about 80% in mice subjected to LPS injection within 12 hours and continued to decline sharply starting from 12 hours after sepsis, reaching almost 42% by 120 hours after LPS injection. Intravenous administration of 30 *μ*g/kg and 50 *μ*g/kg DEX extended lifetime and increased survival rate compared with the LPS group (*p* = 0.038 and *p* = 0.042). Kidney function was evaluated based on serum Cr and BUN which are the primary indicators of the severity of kidney damage. Levels of BUN and Cr in mice kidneys were significantly higher in the LPS group than in the control group, whereas treatment with DEX decreased serum Cr and BUN levels compared to the LPS group (Figure 1(b)). Furthermore, histological analysis in HE and PAS revealed that mice treated with LPS exhibited severe renal pathological lesions, indicated by widespread tubular necrosis, tubular degeneration, cellular swelling, and inflammatory cell infiltration in renal tissues, whereas DEX treatment significantly reversed these effects (Figure 1(c)). Kidney histology scores in the LPS group were significantly higher than those in the control group, whereas kidney scores in the DEX+LPS group were significantly lower than those in the LPS group. Our results suggest that treatment with DEX protects mice from acute kidney injury caused by LPS.

### 3.2. DEX Alleviates Oxidative Stress and Nitrosative Stress in AKI Induced by LPS

**Figure 2:**
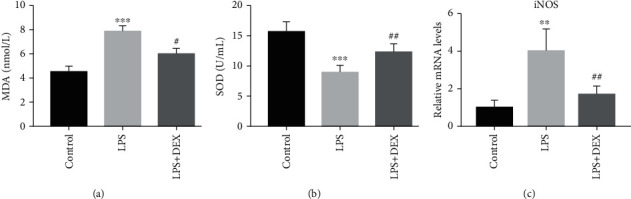
Dexmedetomidine alleviates oxidative stress in AKI induced by LPS. (a) MDA assay in kidney tissues of mice. (b) SOD assay in kidney tissues of mice. (c) Real-time PCR of iNOS in kidney tissues of mice (^∗^*p* < 0.05, ^∗∗^*p* < 0.01, and ^∗∗∗^*p* < 0.0001 versus control; ^#^*p* < 0.05 and ^##^*p* < 0.01 versus LPS. Data were presented as mean ± SEM, *n* = 6). MDA: malondialdehyde; SOD: superoxide dismutase; iNOS: inducible nitric oxide synthase.

AKI induced by LPS breaks intracellular redox balance inducing oxidative stress [16]. MDA concentrations were substantially increased in mice injected with LPS and SOD enzyme activity concentrations were decreased, but treatment with DEX reduced levels of MDA and elevated SOD which had been changed by LPS (Figures 2(a) and 2(b)). Expression levels of mRNA for iNOS in renal tissues were further evaluated. mRNA expression levels for iNOS were considerably upregulated in renal tissues of LPS-treated mice in comparison with the control group. However, DEX remarkably reduced the upregulated mRNA expression levels for iNOS in renal tissues of LPS-treated mice. Our results suggest that DEX considerably alleviates oxidative and nitrosative stress.

### 3.3. DEX Reduced Apoptosis and Expression of p75NTR in Mouse Model of AKI

**Figure 3:**
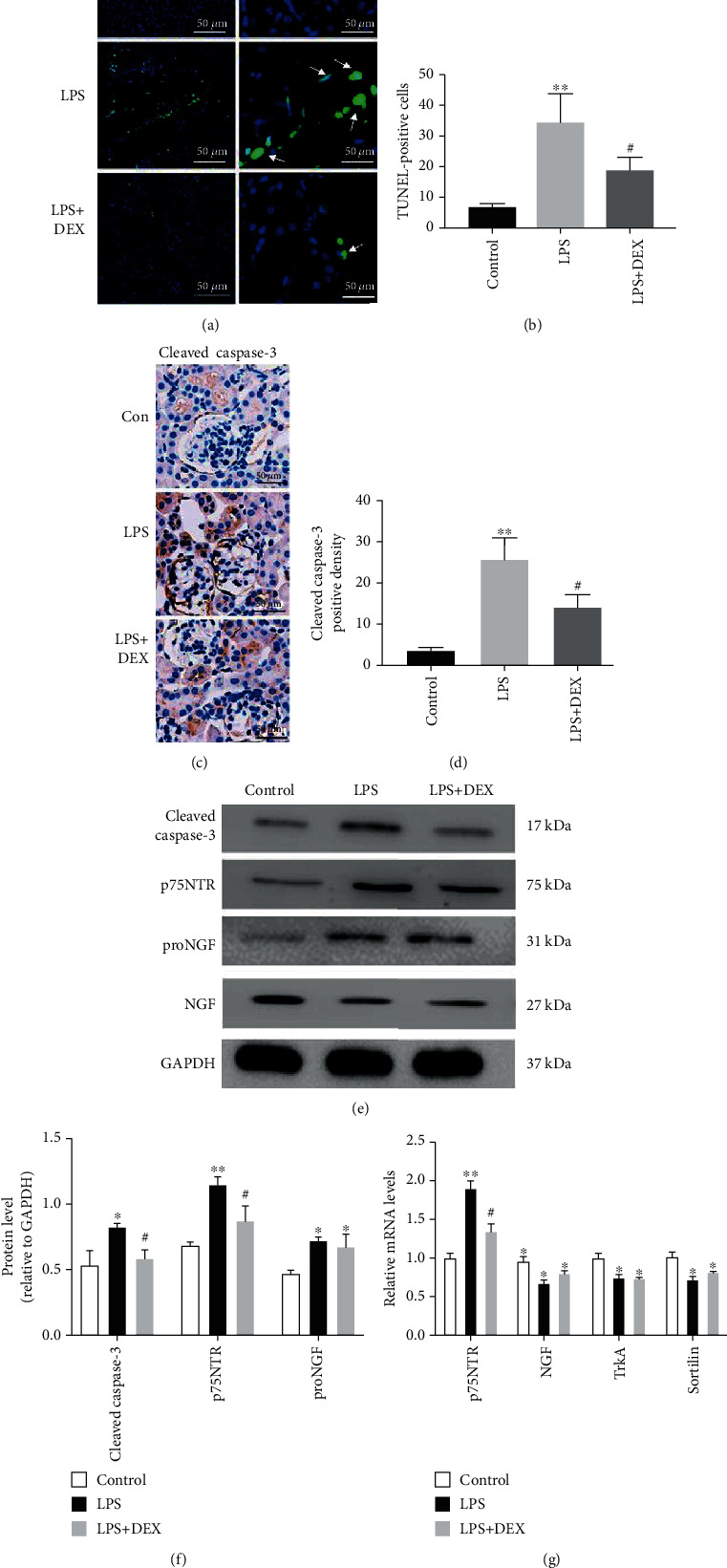
Dexmedetomidine suppressed apoptosis in AKI induced by LPS. (a, b) TUNEL staining in kidney sections of mice (scale bar = 50 *μ*m), white arrows to show TUNEL-positive nuclei and quantitation of TUNEL-positive cells. (c, d) Cleaved caspase-3 staining in kidney sections of mice (scale bar = 50 *μ*m), and quantitation of Cleaved caspase-3-positive cells. (e, f) Western blot analysis and quantitative data for Cleaved caspase-3, p75NTR, proNGF, and NGF in kidney tissues of mice; GAPDH was used as a loading control. (g) p75NTR, NGF, TrkA, and Sortilin gene expression in the kidney tissues of mice (^∗^*p* < 0.05 and ^∗∗^*p* < 0.01 versus control; ^#^*p* < 0.05 and ^##^*p* < 0.01 versus LPS. Data were presented as mean ± SEM, *n* = 6).

A TUNEL assay was performed to examine the effects of LPS and DEX on cell apoptosis (Figure 3). The number of TUNEL-positive cells was significantly higher in mice injected with LPS than in mice in the control group, and fewer positive cells were recorded in the DEX+LPS group than in the LPS group (Figures 3(a) and 3(b)). Our findings indicate that DEX is capable of suppressing LPS-induced apoptosis in the AKI model.

We analyzed expressions of Cleaved caspase-3, p75NTR, and its upstream molecule proNGF to unravel the signaling pathways associated with protective effects of DEX in LPS-induced renal injury. A few Cleaved caspase-3-positive cells were detected in the control group. Nevertheless, the positive density of Cleaved caspase-3 in mice injected with LPS was significantly higher than that of mice in the control group. Pretreatment with DEX substantially decreased the positive density of Cleaved caspase-3 induced by LPS (Figures 3(c) and 3(d)). Western blot results demonstrated that LPS caused significant increase in the levels of Cleaved caspase-3, p75NTR, proNGF, and NGF in kidney tissues compared to the control group (Figure 3). However, levels of Cleaved caspase-3 and p75NTR in kidney tissues of mice treated with LPS were remarkably reduced after pretreatment with DEX. On the contrary, expression of proNGF and NGF remained unchanged after treatment with DEX. Then, we used PCR to complete western blot analysis; as shown in Figure 3(f), the gene expression of NGF and its receptors Sortilin and TrkA decreased in AKI model but remained unchanged after treatment with DEX. These results imply that p75NTR may be involved in pathophysiological processes related to DEX alleviating effects in AKI apoptosis.

### 3.4. Suppression of Apoptosis and ROS Generation in LPS-Induced Cells by Dexmedetomidine and Counteraction through Overexpression of p75NTR

**Figure 4:**
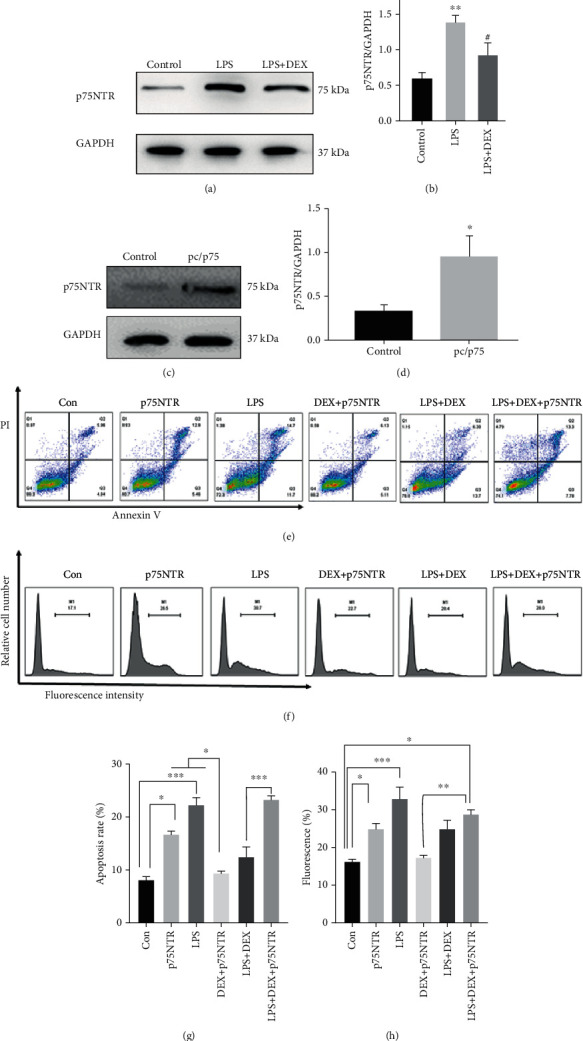
Suppression of apoptosis and ROS generation by dexmedetomidine in cells treated with LPS was reversed by overexpression of p75. (a) Western blots and (b) their semiquantitative analyses of p75NTR in the LPS-treated group or the LPS+DEX-treated group; (c) western blots and (d) their semiquantitative analyses of p75NTR in pc/C-transfected group or pc/p75-transfected group; (e) the apoptotic rate of HK-2 cells was determined by flow cytometry; (g) statistical analysis of the apoptotic rate of HK-2 cells; (f) ROS levels of HK-2 cells was determined by flow cytometry; (h) statistical analysis of the ROS fluorescence (^∗^*p* < 0.05 and ^∗∗^*p* < 0.001 versus control; ^#^*p* < 0.05 and ^#^*p* < 0.01 versus LPS. Data were presented as mean ± SEM, *n* = 6).

In AKI animal model, p75NTR protein is upregulated in the kidney tissue and reversed by DEX pretreatment. Consistently, similar alterations of p75NTR expression were observed in HK-2 cells (Figures 4(a) and 4(b)). Then, HK-2 cells were transfected through vector-mediated overexpression of pc/p75 or a controlled vector and assessed using western blot assay. Expression levels of p75NTR were much higher in pc/p75-transfected HK-2 cells than in pc/C-transfected HK-2 cells (Figures 4(c) and 4(d)).

To investigate whether p75NTR can reverse the protective effect of DEX *in vitro*, HK-2 cells were treated with LPS for 24 hours and flow cytometry assays used to analyze ROS production. ROS generation was significantly increased after LPS stimulation, and treatment with 100 *μ*M of DEX reduced ROS levels which had risen after treatment with LPS. Conversely, ROS levels were considerably higher in pc/p75-transfected cells than in pc/C-transfected cells (Figure 4(h)). Consequently, results indicated that DEX suppressed ROS generation in cells treated with LPS which was countered by overexpression of p75NTR.

Cell apoptosis was substantially promoted in mice treated with LPS, and injection with 100 *μ*M of DEX reduced cell apoptosis from 24.6% to 13.28%. However, antiapoptotic effects of DEX were reversed through overexpression of p75NTR. Overexpression of p75NTR reversed the protective effects of DEX on apoptosis induced by LPS in HK-2 cells (Figure 4(g)).

### 3.5. Inhibition of p38 Mitogen-Activated Protein Kinase- (MAPK-) Jun N-Terminal Kinase (JNK) Signaling Pathway by DEX in HK-2 Cells

**Figure 5:**
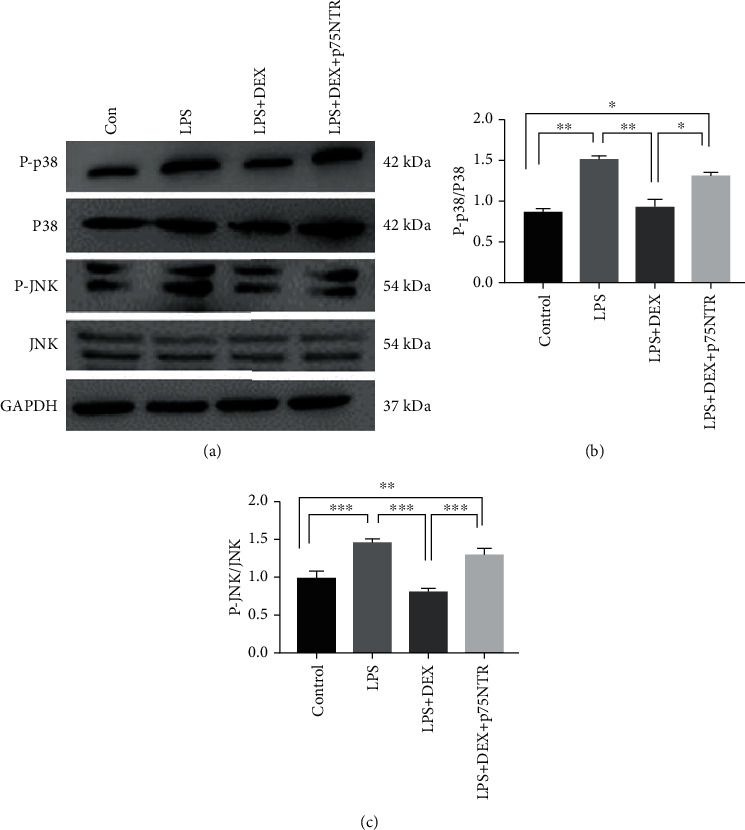
Inhibition of the p38MAPK-JNK signaling pathway by dexmedetomidine in HK-2 cells of AKI which was reversed by overexpression of p75NTR. (a) Representative western blots and (b, c) their semiquantitative analyses (^∗^*p* < 0.05 and ^∗∗^*p* < 0.001 versus control; ^#^*p* < 0.05 and ^##^*p* < 0.01 versus LPS. Data were presented as mean ± SEM, *n* = 6).

The increased proNGF mediated p75NTR activation and subsequently induced the activation of p38MAPK and JNK signaling [33]. ROS has been reported to activate proapoptotic signaling pathways such as JNK and p38MAPK through stimulation of upstream kinases [34]. Western blot analysis revealed that both JNK and p38MAPK pathways were activated by LPS (Figure 5). Incubation of HK-2 cells with DEX 24 hours significantly reduced expression of phosphorylated protein levels in p38MAPK and JNK1/2 pathways. Notably, p75NTR inhibited phosphorylation of JNK and p38MAPK pathways after treatment with DEX. Overall, the p75NTR/JNK/p38MAPK axis is involved in apoptosis associated with LPS.

## 4. Discussion

Acute kidney injury is one of the most severe complications of sepsis and is a rapid renal dysfunction associated with inflammation and oxidative stress. Intraperitoneal injection of LPS to induce sepsis is a commonly used animal model. LPS is a classic TLR4 agonist which can induce an immediate and robust inflammatory response thus stimulating activation of the innate immune system in human sepsis [3]. The most significant advantage of this model is that the technology used is simple and easy to replicate. Our results revealed that treatment with DEX significantly alleviated LPS-induced oxidative stress and apoptosis which consequently attenuated kidney dysfunction. Overexpression of p75NTR enhanced apoptosis, ROS generation, and phosphorylation of p38MAPK-JNK pathway but eventually reversed the protective effects of DEX in sepsis-associated AKI.

DEX is a class of highly selective *α*_2_-adrenergic receptor agonists with receptors which are widely distributed in the proximal and distal tubules of the kidney [8]. The protective effects of DEX have been reported against oxidative stress, apoptosis, and pyroptosis in the brain and peripheral tissues in various *in vitro* and *in vivo* models [35, 36]. Experimental studies of animals have indicated that DEX attenuates LPS-induced renal dysfunction and histological tissue damage in the kidneys [37]. Oxidative stress has been identified as one of the critical contributors of pathogenesis in AKI [6]. Production of free radicals is eliminated by intracellular antioxidant enzymes such as SOD [38], thus maintaining the balance between production and elimination of free radicals. AKI increases superoxide production and inhibits SOD activity. MDA is the end product of lipid peroxidation and its production is increased in kidney tissues after renal I/R injury [38]; MDA contributes to cell apoptosis and is strongly involved in AKI [39]. In addition, we established that DEX reduced markers of oxidative stress including MDA and SOD and apoptosis as indicated by TUNEL and Cleaved caspase-3 analysis. The kidney consists of tubules, renal vesicles, and glomeruli. Studies have reported that sepsis-related AKI is principally caused by glomerular or tubular apoptosis [7]. In support of animal studies, our *in vitro* data firstly demonstrated that DEX alleviated LPS-induced ROS production and apoptosis in the kidney tissue, suggesting that the inhibitory effect of DEX in sepsis-related AKI is partially due to alleviation of oxidative stress and apoptosis in the tubules.

p75NTR is a member of tumor necrosis factor receptor superfamily and a transmembrane receptor that can transduce a “death”—the receptor-mediated apoptotic cascade in several neuronal populations [40]. Several studies have revealed that silencing p75NTR prevents cell death and oxidative stress, therefore alleviating diseases like neurodegenerative disorders and microvascular degeneration [41, 42]. Other than its abundant expression in the central nervous system, p75NTR can also be expressed in the kidneys [27]. During the kidney development, all the developing glomeruli show a marked increase of p75NTR expression during the differentiation of mesenchymal into podocytes [43]. Previous studies have reported the upregulation of p75NTR in a mouse model with unilateral ischemic reperfusion injury [44]. Strikingly, p75NTR RNA silencing also inhibits human renal cell carcinoma (ACHN) cell migration implying that p75NTR may play an essential role in kidney diseases [45]. Nevertheless, its fundamental role in kidney injury remains unknown. In the present study, p75NTR was considerably expressed in kidney tissues in mice with LPS-induced AKI, accompanied by increased expression of markers of apoptosis and oxidative stress. These results indicate a positive correlation of p75NTR with apoptosis and oxidative stress in sepsis-related AKI. Previous studies [45] have shown that knocking down p75NTR can inhibit the ACHN cell migration and therefore attenuate the kidney injury. In our study, DEX decreased ROS production and apoptosis upon LPS treatment in HK-2 cells. However, overexpression of p75NTR can abolish the protective effects of DEX. These findings suggest that the protective effect of DEX is mediated by p75NTR signaling, at least in part.

In the animal studies, we have observed that LPS treatment increased the upregulation of proNGF and p75NTR. The increased ligand can act on the increased p75NTR and mediate the downstream signaling. However, DEX cannot inhibit the upregulated proNGF upon LPS treatment. Therefore, we did not examine the proNGF level in our further studies. Notably, our study cannot identify if the effect of p75NTR is ligand dependent or not. Given that DEX can downregulate the expression of p75NTR, its protective effect may not be related to the proNGF level. However, other ligands including proBDNF and proneurotrophin-3 may be changed after LPS and/or DEX treatment which need to be studied in the future.

Studies have revealed that p75NTR induces apoptosis in a JNK-dependent manner [46] and that p38MAPK and JNK pathways are crucial for amyloid-induced cell death that is mediated by p75NTR [47]. The activity of JNK can be upregulated by neurotrophins such as proNGF and proBDNF under apoptotic conditions with p75NTR [45, 48]. Currently, there is no direct evidence that DEX ameliorates septic AKI via the p38MAPK-JNK signaling pathway. Nonetheless, Walshe et al. [49] reported that DEX protects the kidney and other organs from subsequent I/R damage via p38MAPK-JNK pathway-dependent mechanisms. Targeted inhibition of p38MAPK pathway reduces renal cell apoptosis and improves renal function after I/R injury [50]. Furthermore, several studies have reported that ROS can initiate phosphorylation of JNK and p38MAPK pathways [51]. The p38MAPK-JNK pathway has been proven to have an essential function in determining the fate of renal tubular cells [52]. Our results correspond with previous observations and demonstrate that DEX can protect the kidney from oxidative stress and apoptosis by inhibiting expression of p75NTR and subsequent p38MAPK and JNK signaling pathways in tubular epithelial cells stimulated by LPS.

Over the last 30 years, numerous attempts are under way to improve outcomes for AKI patients, including therapy targeted at hemodynamics, diuretics, and oxidative stress, but little progress has been made [53]. Despite early goal-directed therapy (EGDT) becoming a standard therapy in sepsis shock, recent multicenter trials of EGDT failed to show improved survival, as well as a reduction in AKI or inflammation [54]. New therapies for sepsis-associated AKI are urgently needed. To date, large amounts of preclinical researches have investigated several potential targets to treat AKI, such as statins [55], N-acetyl-cysteine [56], and alkaline phosphatase [57], but none of them have translated into human clinical experiments of AKI. DEX is widely used in patients undergoing mechanic ventilation in ICU and operations because of its analgesic and sedative effect [58, 59]. We and other studies [60] have shown that treatment with DEX markedly attenuates LPS-induced renal dysfunction in mice and reduces injury in tubular epithelial cells. These findings point to a potential treatment strategy of DEX in sepsis-associated AKI. The effects of DEX in clinical patients warranted further study in the future.

In summary, our results demonstrated the following: (1) DEX inhibited LPS-induced ROS production and apoptosis in tubular epithelial cells indicating that the inhibitory effects observed after treatment with DEX in mice with sepsis-related AKI are partially due to alleviation of oxidative stress and apoptosis in the tubules; (2) p75NTR was highly expressed during progression of sepsis-related AKI, and overexpression of p75NTR reversed the protective effects of DEX against ROS production and apoptosis in LPS-treated tubular epithelial cells; (3) DEX reduces oxidative stress and apoptosis possibly by a mechanism that may involve the p75NTR, p38MAPK, and JNK pathways in sepsis-associated AKI. Results of this study elucidate the potential protective and molecular mechanisms of DEX in sepsis-related AKI from a perspective of oxidative stress and provide a theoretical basis for future clinical research.

## 5. Conclusions

DEX ameliorated AKI in mice with sepsis by practically reducing oxidative stress and apoptosis through the regulation of p75NTR/p38MAPK/JNK signaling pathways.

## Data Availability

The data used to support the findings of this study are available from the corresponding author upon request.
